# An Overview of the Medicinally Important Plant Type III PKS Derived Polyketides

**DOI:** 10.3389/fpls.2021.746908

**Published:** 2021-10-14

**Authors:** Renu Bisht, Aniket Bhattacharyya, Ankita Shrivastava, Priti Saxena

**Affiliations:** Chemical Biology Group, Faculty of Life Sciences and Biotechnology, South Asian University, New Delhi, India

**Keywords:** type III polyketide synthases, chalcones, alkaloids, chromones, anthrones, xanthones, pyrones, cannabinoids

## Abstract

Plants produce interesting secondary metabolites that are a valuable source of both medicines for human use, along with significant advantages for the manufacturer species. The active compounds which lead to these instrumental effects are generally secondary metabolites produced during various plant growth phases, which provide the host survival advantages while affecting human health inadvertently. Different chemical classes of secondary metabolites are biosynthesized by the plant type III polyketide synthases (PKSs). They are simple homodimeric proteins with the unique mechanistic potential to produce a broad array of secondary metabolites by utilizing simpler starter and extender units. These PKS derived products are majorly the precursors of some important secondary metabolite pathways leading to products such as flavonoids, stilbenes, benzalacetones, chromones, acridones, xanthones, cannabinoids, aliphatic waxes, alkaloids, anthrones, and pyrones. These secondary metabolites have various pharmaceutical, medicinal and industrial applications which make biosynthesizing type III PKSs an important tool for bioengineering purposes. Because of their structural simplicity and ease of manipulation, these enzymes have garnered interest in recent years due to their application in the generation of unnatural natural polyketides and modified products in the search for newer drugs for a variety of health problems. The following review covers the biosynthesis of a variety of type III PKS-derived secondary metabolites, their biological relevance, the associated enzymes, and recent research.

## Introduction

Plants are essential to life forms on Earth, playing a key role in the well-being of both humans and the entire ecosystem. Besides being primary producers for all food chains, plants biosynthesize secondary metabolites that exhibit a wide range of chemical structures and biochemical properties (Compean and Ynalvez, 2014; Freiesleben and Jäger, 2014; Tiwari and Rana, 2015). Polyketides, fatty acids, terpenoids, phenylpropanoids, alkaloids, and several other specialized amino acids and carbohydrates make up the majority of plant secondary metabolites. Plants use secondary metabolites for physiological, survival, and maintenance purposes, as well as for defending against pathogens, fighting off herbivores, protection against UV exposure, and various other biotic and physical stresses. The pharmacological properties of secondary metabolic compounds are most responsible for plants bearing medicinal and therapeutic properties (Tiwari and Rana, 2015). This review highlights the medicinal, pharmaceutical, and industrial properties of polyketide secondary metabolites, with an emphasis on their ability to assist with biological processes.

Several chemically distinct classes of secondary metabolites are biosynthesized by the type III polyketide synthases (PKSs). Type III PKSs were first discovered and characterized from plants and are now known to occur in many different life forms. Structurally, type III PKSs are small homodimeric proteins with each monomer containing an independent active site comprising of a *Cys, His, Asn* catalytic triad. Type III PKSs typically utilize a simple chemical strategy of initial priming with a monocarboxyl-coenzymeA (CoA) starter substrate that undergoes repetitive decarboxylative condensations with simple dicarboxyl-CoA extender substrate to generate a poly-β-keto intermediate that can either be cyclized or released as a linear product. Many variations are possible when dealing with these proteins because they have the capacity to accept several different starters and extender substrates and conduct a variety of condensation and elongation reactions. Through these various modes of ring closure, they enable a wide range of product profiles. By post-synthesis modifications, the polyketide core further evolves into distinct bio- functionalities (Abe, 2012).

**Figure 1:**
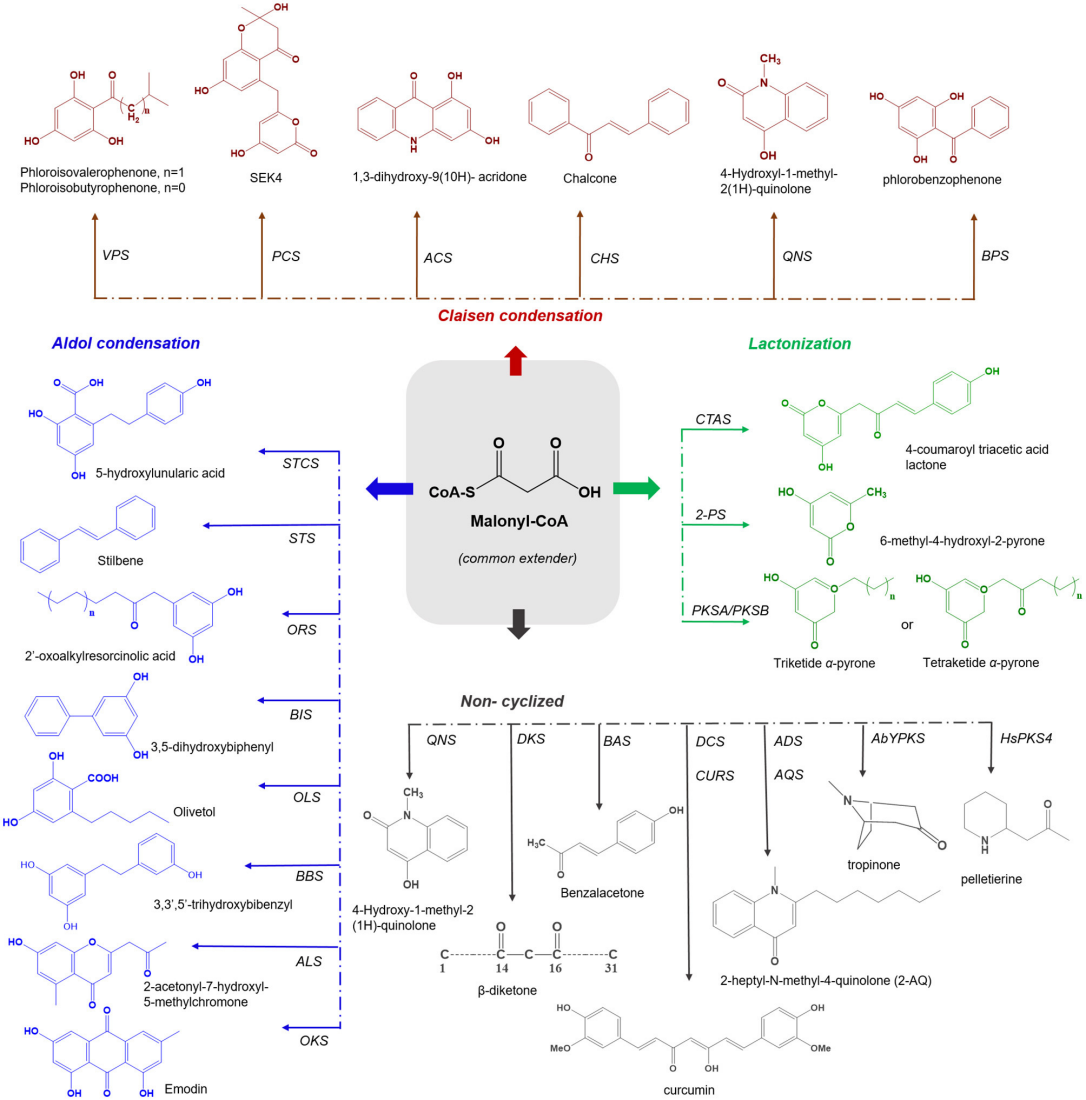
Overview of different cyclization governed by plant type III PKSs. The common extender malonyl-CoA is utilized by several plant type III PKSs. The C6-O-C1 Claisen condensation cyclized polyketides are shown in red color (on the top), C7-O-C2 aldol condensation cyclized polyketides are depicted in blue (left), the C5-O-C1 lactonization type cyclized products are shown in green color (right) and the non-cyclized polyketides are presented in gray (bottom). VPS, valerophenone synthase; PCS, pentaketide chromone synthase; ACS, acridone synthase; CHS, chalcone synthase; QNS, quinolone synthase; BPS, benzaphenone synthase; STCS, stilbene carboxylate Synthase; STS, stilbene synthase; ORS, 2′-oxoalkylresorcinol Synthase; BBS, bibenzyl synthase; OLS, Olivetol synthase; BIS, biphenyl synthase; ALS, aleosone synthase; OKS, Octaketide synthase; QNS, quinolone synthase; DKS, diketide synthase; BAS, benzalacetone synthase; DCS, diketide-CoA synthase; CURS, curcumin synthase; ADS, alkyldiketide-CoA synthase; AQS, alkylquinolone synthase; AbYPKS-type III PKS from *Atropa belladonna*; HsPKS-type III PKS from *Huperzia serrata*; CTAS, p-coumaroyl triacetic acid synthase; 2-PS, pyrone synthase and PKSA and PKSB, anther specific chalcone like synthase and PKSB.

Plant type III PKSs accept a wide variety of starter substrates, including intermediates of the phenylpropanoid pathway, CoA/N-acetyl cysteamine (NAC), and activated aliphatic/aromatic mono- and di-carboxylic acids. A tetraketide intermediate is produced with three rounds of condensation of the starter unit with the extender substrate. However, in some cases, this number can rise to eight or more rounds of condensation (Abe et al., 2004). Type III PKSs cyclize the reaction intermediates using three distinct ring-folding chemistries; C_6_-C_1_ Claisen condensation exemplified by the chalcone synthases (CHSs), C_2_-C_7_ aldol condensation unique to the stilbene synthases (STSs), and C_5_-O-C_1_ lactonization that results in derailment products observed in certain cases (Figure 1) (Shimizu et al., 2017).

**Figure 2:**
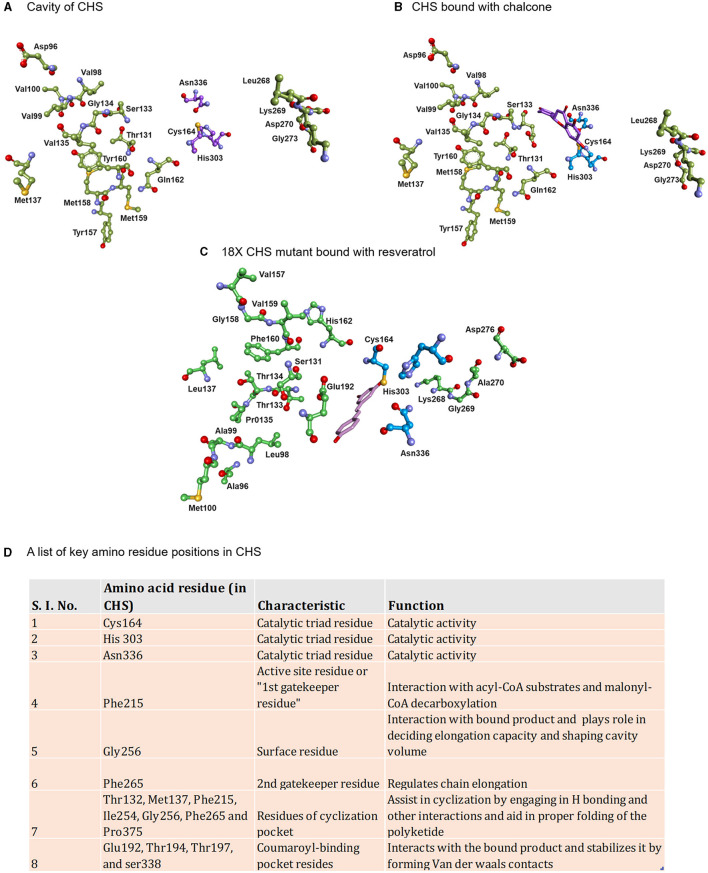
Displaying the active site lining residues of **(A)** prototype chalcone synthase **(B)** CHS (docked with chalcone) and **(C)** 18X mutant of CHS (docked with resveratrol). The key amino residues of CHS are shown in the table along with the models **(D)**.

These proteins, surprisingly, show differences in starter specificity, extension count, and final product cyclization. The structural studies have identified several key amino acid residues affecting the starter specificity, cavity volume, rounds of chain elongation, and cyclization (Figure 2). This produces variations in the products with slight alterations in biological activity (Abe and Morita, 2010; Morita et al., 2019). Type III PKSs have attracted a lot of attention because of their structural and mechanistic simplicity and their ability to produce a broad array of important secondary metabolites (Dibyendu, 2015). Despite the exhaustive research that was carried out, which led to the discovery of numerous crystal structures of plant Type III PKSs and to the identification of crucial residue positions that determine the structure of the final product, the exact mechanism behind the cyclization preference remains unknown. In this study, we aimed to classify these polyketide metabolites based on the mode of cyclization for their biogenesis while addressing their biological significance together with commenting upon the biosynthetic potential, limitation, and uniqueness of the synthesizing enzymes. In this review, the emphasis is on the biological and pharmaceutical strength of the polyketide-derived secondary metabolites while highlighting the mechanism of the biosynthetic Type III PKSs. Since secondary metabolites sourced from plants are currently the primary compounds used for drug development and are frequently utilized to address numerous health concerns, we believe that our review will provide a welcome boost to the status quo of these fundamental polyketide metabolites.

## Different Modes of Cyclization of Plant Type III PKSs and Corresponding Polyketides

Type III PKSs are well-equipped in generating chemo-diversity from a limited pool of precursor molecules, by controlling the number of condensation and directing the cyclization of the common intermediate to generate different classes of the final product. In this review, type III PKS derived secondary metabolites are categorized into four different groups based on the mode of cyclization; (i) C_6_-C_1_ Claisen, (ii) C_2_-C_7_ aldol, (iii) C_5_-O-C_1_ lactonization, and (iv) Non-cyclized in addition to discussing their biological activities. An overview of the different cyclization types, corresponding enzymes, and their products are presented in Figure 1.

### C_6_-C_1_ Claisen Cyclization Derived Type III PKS Products

#### Chalcone Synthase—Chalcones, Aurones, and Flavonoids

**Figure 3:**
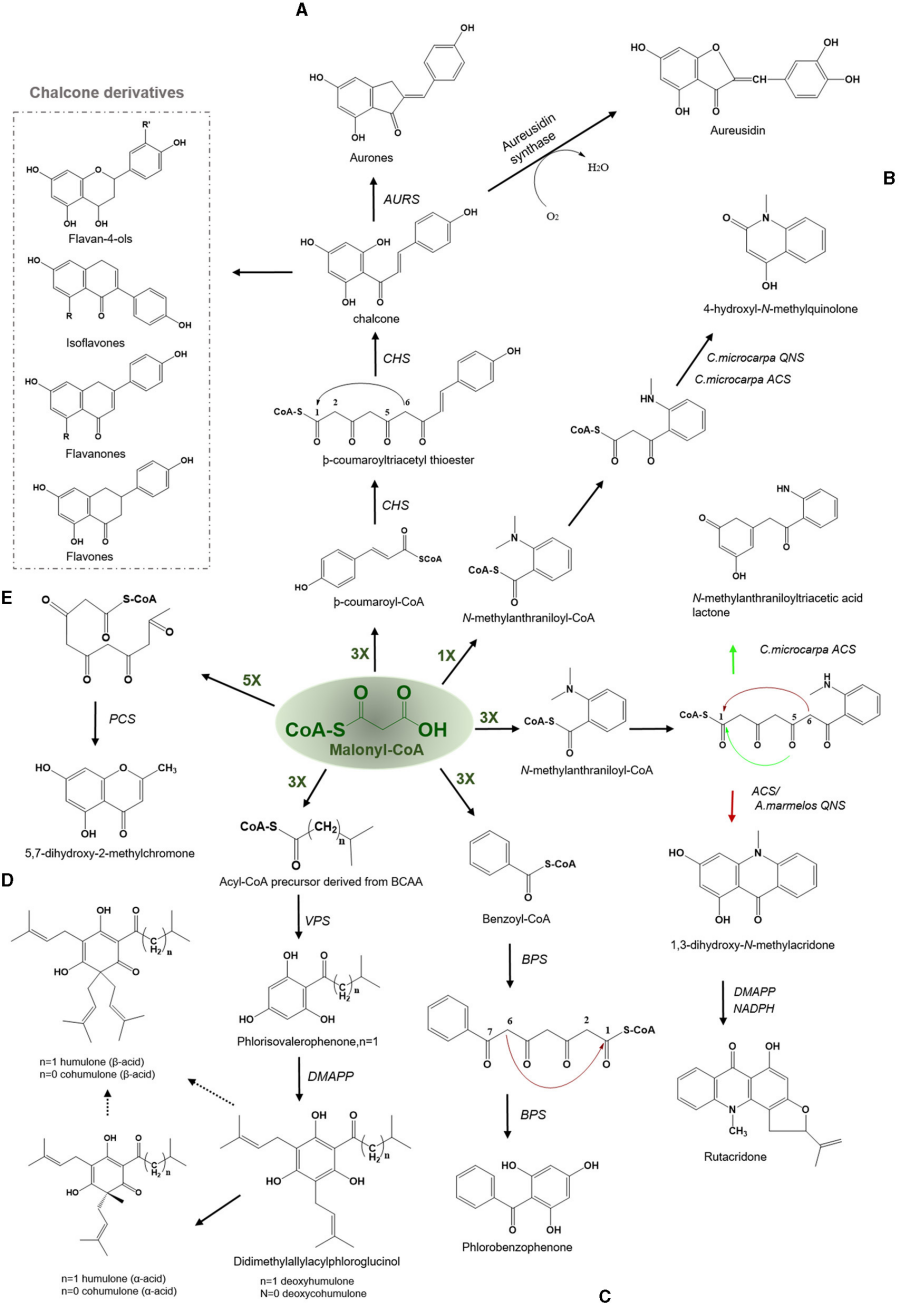
Examples of Claisen type condensation reactions employed by various type III PKSs to produce a variety of polyketide products. Describes the reaction catalyzed by **(A)** chalcone synthase (CHS) and Aureusidin synthase, **(B)** acridone synthase (ACS), **(C)** benzophenone synthase (BPS), **(D)** valerophenone synthase (VPS), and **(E)** pentaketide chromone synthase (PCS). The dashed arrows represent the proposed pathways. The green-colored nX value represents the number of malonyl-CoA molecules employed in a reaction; DMAPP, dimethylallylpyrophosphate; BCAA, branched-chain amino acid.

The enzyme CHS is critical to the production of chalcone, a vital compound in the synthesis of flavonoids in plants. The CHS is ubiquitously present in almost all plant species (Rammohan et al., 2020). More than 650 *chs*-like gene sequences have been identified in plants since the first *chs* gene was reported in 1983 (Reimold et al., 1983). CHS catalyzes three iterative decarboxylative condensations of the extender malonyl-CoA with starter -coumaroyl-CoA to yield a linear tetraketide, -coumaroyl triacetyl thioester, which undergoes an intramolecular C_6_-C_1_ Claisen condensation based cyclization to produce a chalcone. The chalcone moiety can subsequently give rise to naringenin, a precursor of flavonoids, antimicrobial phytoalexins, and anthocyanins in plants (Figure 3A) (Austin and Noel, 2003). The C_6_-C_1_ Claisen cyclization is a common mode of cyclization found in a variety of plant type III PKSs, such as CHS and similar CHS-like PKSs.

The first crystal structure of CHS2 from alfalfa was solved in 1999, and it revealed insights regarding the basic functioning of the enzyme and highlighted the amino acid residues that are key to selecting starters, thus defining the initiation/elongation cavity of the active site (Ferrer et al., 1999). The key intermediate chalcone possesses numerous bioactivities including antioxidants, antimicrobials, anti-inflammatory drugs, antifungals, cytotoxics, antitumor, and chemopreventives (Dao et al., 2011). Numerous modified chalcones such as hydroxy or/and methoxy-substituted chalcones, methylated, prenylated, geranylated, and other monomeric derivatives, chromeno- and furano-chalcones, dimeric chalcones, and dihydrochalcone have been identified to date with potent biological activities (Rozmer and Perjési, 2016). Aurones [2-benzylidenebenzofuran-3(2H)-ones] which are a new class of naturally occuring flavonoids found in fruits and flower functions as phytoalexins against infections and impart yellow pigmentation to plant parts (Hemmerling and Hahn, 2016). The biosynthesis of aurones is catalyzed by the aurone synthase, a catechol oxidase belonging to the plant phenol oxidase (PPOs) family. Interestingly, PPO is a chalcone-specific enzyme and has specificity toward the chalcone intermediate (Nakayama et al., 2001). Aurones are synthesized similarly from -coumaroyl-CoA and malonyl- CoA *via* tetrahydroxychalcone and trihydroxychalcone using the CHS and chalcone reductase (Figure 3A). Subsequently, trihydroxychalcone is converted to aurones by the action of the aureusidin synthase (Motohashi, 2008). Aurones have been reported to possess insect antifeedant, anticancer, antiparasitic, antileishmanial, and antifungal activities. Also, they can act as tyrosine inhibitors and antioxidants. Aureusidine, a common aurone, is an iodothyronine deiodinase inhibitor, an enzyme that participates in the synthesis and regulation of thyroid hormones. Some synthetic aurones can be used as potential cancer chemotherapeutic agents, due to their binding affinity with the nucleotide-binding domain of P-glycoprotein to inhibit the cyclin-dependent kinases in connection with anti-proliferative activities (Zwergel et al., 2012). *Uvaria hamiltonii* derived aurones which are known tubulin-binding agents have also been evaluated for their anticancer activity (Lawrence et al., 2003).

The products of CHS-derived pathways have compelling biological and medicinal properties. Among them, flavonoids, are the largest class of phenolic compounds in higher plants and bryophytes (Jiang et al., 2006; Yu et al., 2015) which constitutes around 6000 structurally diverse flavonoids including chalcones, flavones, flavonols, flavandiols, anthocyanins, and proanthocyanidins or condensed tannins and aurones, which are present only in few plant species (Austin and Noel, 2003). These polyketide derivatives display varied biological and physiological activities, such as plant-pollinator attractors (flower pigments), UV protectors, phytoprotectants, and feeding deterrents against insects and mammals (Harborne and Williams, 2000; Dao et al., 2011). Isoflavones, in particular, demonstrate various activities, including potent phytoestrogen, antiangiogenic, antioxidant, and anticancer (Figure 3A) (Kumar and Pandey, 2013; Vitale et al., 2013). Especially, isoflavones such as daidzein, and coumestrol, have potent estrogenic activity. Certain isoflavones have various disease-preventive benefits, including reducing the risk of osteoporosis and preventing postmenopausal conditions and cardiovascular diseases (Khare and Katiyar, 2012). The *in planta* role of flavonoids, includes intracellular and extracellular signaling, male fertility agents, allelochemicals, and defense molecules (Samanta et al., 2011). In addition, they play a critical role in the nodulation, pollen fertility, auxin transport, and coloration of flowers as a visual signal for attracting pollinators (Kootstra, 1994; Mol et al., 1998; Feild et al., 2001; Mierziak et al., 2014). They are also responsible for protecting leaf cells against photo-oxidative damage and increasing nutrient recovery efficiency during senescence. Flavonols contribute to the stress response of plants and are the most ancient and widespread flavonoids to date (Ghasemzadeh and Ghasemzadeh, 2011). Interested readers are directed to the review by Falcone Ferreyra et al. (2012) which deals extensively with biosynthesis, versatility, and biotechnological significance of flavonoids.

#### Acridone Synthase-Acridone Alkaloids

**Figure 4:**
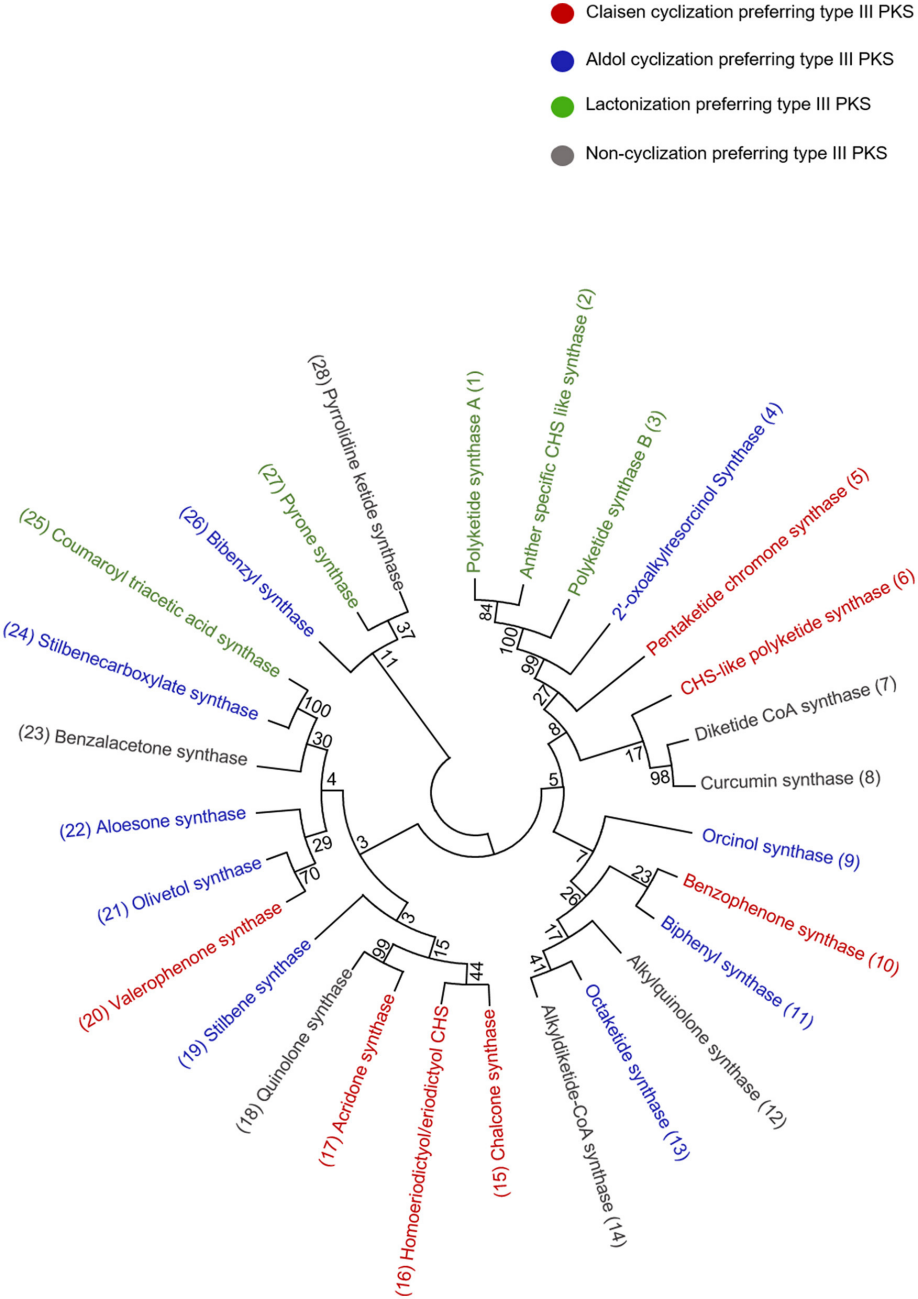
Phylogenetic tree analysis of the representative type III PKSs from each cyclization class; the alignment was generated by CLUSTALW. The examples of each enzyme class are color-coded and the tree was constructed using the Maximum likelihood method by using 1,000 bootstraps.

Acridone alkaloids were first found in plants in 1948 and were known since the turn of the century (Vasil, 2012). However, the occurrence of acridone alkaloids is restricted to the *Rutaceae*, a medicinal plant family. *Ruta graveolens* produced 14 different acridones that were isolated from the culture suspension along with four other isolated from *Ruta* species and *Boeinninghausenia albiflora* (Baumert et al., 1994). Various monomeric acridones and coumarin-acridone dimers have been isolated from citrus plants. Acridones are anthranilate-derived alkaloids and biosynthesized by polyketide pathways in plants. The acridone synthase (ACS) has been characterized from *R. graveolens*. It catalyzes the reaction similar to CHS, but rather uses *N*-methylanthraniloyl-CoA as a starter substrate and performs the decarboxylated condensation of three malonyl-CoA units followed by the Claisen condensation to produce 1,3-dihydroxy-*N*-methylacridone, a common intermediate which leads directly to the formation of more complex acridones such as rutacridone (Figure 3B) (Lim et al., 2016). Despite, 74% amino acid sequence similarity between *R. graveolens* ACS and CHS, ACS differ in substrate specificity and fail to efficiently utilize -coumaroyl-CoA, the common CHS starter. The difference in the cavity volume which is bigger in ACS accounts for the differences in starter specificity and ability to accommodate larger *N*-methylanthraniloyl-CoA as starter molecules (Springob et al., 2000). A recently characterized Type III PKS, quinolone synthase (QNS), depicts similar substrate specificity as ACS and is involved in the formation of quinolone alkaloids in *Aegle marmelos* (Resmi et al., 2013). Both ACS and QNS share similar substitutions (T132S, S133A, and F265V) in the cavity lining residue sites compared with CHS, which might account for their unique substrate specificity (Lukačin et al., 2001). Other *Citrus microcarpa* derived ACS shows remarkable substrate promiscuity and is employed in the production of 4-hydroxy-*N*-methylquinolone, 1,3-dihydroxy-*N*-methylacridone, and *N*-methylanthranilriacetic acid lactone in addition to producing chalcone, benzophenone, and phloroglucinol scaffolds (Mori et al., 2013). The crystal structure analysis of both ACS and QNS of *Citrus microcarpa* has revealed the presence of a wide active site entrance that explains the promiscuous behavior of these enzymes. These results provided the first structural basis for the generation of anthranilate-derived alkaloids by Type III PKSs. One of the examples of similar acridone synthesizing Type III PKS comes from a non-Rutaceae family plant, *Huperzia serrata* (Chinese club moss) which also exhibits similar promiscuity of the substrate and produces several different chemical scaffolds from the same enzymatic core. PKS1 of *H. serrata* shares 44% sequence identity with other members of the CHS superfamily and groups with the non-CHS Type III PKSs [Figure 4 (6)]. PKS1 also contains a large active site that provides access to bigger starter molecules that allows the synthesis of aromatic tetraketides such as chalcones, benzophenone, phloroglucinols, and acridones (Wanibuchi et al., 2007). Functionally, acridone molecules have several important biological activities and the basic acridone skeleton can be further modified by prenylation by the action of dimethylallyldiphosphate (DMAPP), followed by the formation of an additional heterocylic five- or six-membered ring. Due to their planar structure acridones can intercalate DNA and therefore lead to genotoxicity (Michael, 2001). Acronycine and its derivative from *Acronychia baueri* showed potential anticancer activity and the derivative, benzoacronycine has DNA methylation activity and is currently a subject of phase I clinical trials (Nguyen et al., 2009). In a cytotoxic potency analysis of two acridones alkaloids and various other furanacridones, the acridone alkaloid, arborinine, displayed the the best activity with all the three tested human cancer cell lines viz., HeLa, MCF7, and A431 (Rethy et al., 2007). Other activities of acridones are being antimicrobial, antimalarial, antifeedant, and inhibitors of acetylcholinesterase (Yang et al., 2013; Gensicka-Kowalewska et al., 2017).

#### Benzophenone Synthase—Benzophenones and Xanthones

Benzophenone is a class of plant secondary metabolites biosynthesized by the benzophenone synthase (BPS). BPS display a substrate specificity toward smaller starters such as benzoyl-CoA and its derivatives. It catalyzes the decarboxylative condensation of three molecules of malonyl- CoA with benzoyl-CoA to yield a tetraketide intermediate 2, 4, 6-trihydroxybenzophenone (Figure 3C). The BPS together with the biphenyl synthase (BIS) forms a separate clade with non-CHS like PKSs in the phylogenetic tree [Figure 4 (10) and (11)]. Despite sharing 60% sequence identity with CHS, BPS differs in starter specificity. The crystal structure studies of BPS from *Hypericum androsaemum* highlighted a mutation in the active site of the enzyme that hinders its ability to utilize larger substrate molecules thereby only accepting small hydrophobic molecules such as benzoyl-CoA (Stewart et al., 2017). Interestingly, CHS triple mutant (L263M/F265Y/S338G) produced an enzyme that preferred benzoyl-CoA over -coumaroyl-CoA as a substrate (Liu et al., 2003). The 2, 4, 6-trihydroxybenzophenone, an intermediate in the BPS reaction gives rise to various chemical products with diverse biological activities. The BPS from the medicinal herb *Centaurium erythraea* utilizes 3-hydroxybenzoyl-CoA as a preferred starter substrate to produce 2, 3′, 4, 6- tetrahydroxybenzophenone, the precursor of xanthones.

The prenylated xanthones (α-mangostin) and other xanthones have antimicrobial, antituberculosis, antioxidant pro-apoptotic, anti-inflammatory, antidiabetic, and CNS stimulation activities (Pinto et al., 2005; Nualkaew et al., 2012; Negi et al., 2013).

#### Valerophenone Synthase—Valerophenone, Deoxy-Humulone, and Cohumulone

Valerophenone is butyl phenyl ketone which is an intermediate in the production of α and β-acids in the lupulin gland of the agriculturally important hop plant (*Humus lupus* L.). The α and β-acids are responsible for the bitter taste and aroma of the beer along with its stability. Valerophenone is biosynthesized by valerophenone synthase (VPS), a homolog of CHS that catalyzes a typical CHS-like reaction but differs in starter selectivity since it uses the branched-chain isobutryl-CoA or isovaleryl-CoAs as starter substrates. The decarboxylative condensation of three molecules of malonyl- CoA with these starter molecules yields phlorobutrylophenone or phloroisovalerophenone, respectively, which further gives rise to deoxyhumulone and deoxycohumulone. Deoxyhumulone and deoxycohumulone subsequently oxidize to yield α and β-acids (Okada and ITo, 2001) (Figure 3D). In addition to the flavor of a beer, the hop-derived secondary metabolites polyphenols, essential oils and resins have various pharmacological and biological activities such as estrogenic, antioxidant, anti-inflammatory, antimicrobial, and anti-cancer. Moreover, the α-bitter acids from hop have also been associated with combating lifestyle diseases (Karabín et al., 2016).

#### Pentaketide Chromone Synthase—Chromones

Pentaketide chromone (5,7-dihydroxy-2-methylchromone) is biosynthesized by a unique type III PKS, known as the pentaketide chromone synthase (PCS) that catalyzes the condensation of five molecules of malonyl-CoA as starter substrates (Figure 3E). The substitution of a single amino acid residue, Met207 with Gly, in PCS (corresponding to the Thr197 residue in the active site of msCHS) produced an enzyme that efficiently generates aromatic octaketides, SEK4 and SEK4b (Wanibuchi et al., 2011). PCS from *Aloe arborescens* display promiscuous substrate specificities; however, the enzyme only produced triketides and teraketides α-pyrones, accepting a large number of starter molecules, from aromatic to aliphatic-CoA esters (Abe et al., 2005). PCS shares 50–60% sequence identity with the CHS superfamily of enzymes. Additionally, it shows 50% identity with ALS2c, from *Rheum palmatum* which catalyzes the formation of a heptaketide, aloesone (2-acetonyl-7-hydroxy-5-methylchromone), by condensing one molecule of acetyl-CoA with six molecules of malonyl-CoA. *A. arborescens* is well-known to be a rich source of chromones and anthraquinones and PCS is involved in the biosynthesis of many of these chromone molecules.

Chromones are distributed in virtually every known terrestrial plant and more than 4,000 natural derivatives have been isolated and structurally elucidated until now. They are three ringed phenolic compounds and usually contribute to plant defenses and are one of the most extensively studied bioactive compounds. They have outstanding biological activities, including antimicrobial, antiviral, anticancer, anti-inflammatory, and antioxidant. They are essentially divided into three subgroups, namely simple chromones, pyranochromones, and furanochromones. Cytotoxic and antimicrobial compounds are simple chromones, like glucoside biflorin and 2-phenoxychromone, capillarism. Khellin and visnagin (dehydrokhelline) are two phototoxic and vasorelaxant cAMP phosphodiesterase inhibitors derived from *Ammi visnaga* (Apiaceae) seeds (Polya, 2003). It is interesting to note that newer unusual chromones, such as 2-(2-phenylethyl chromone) (PEC), exhibits promising neuroprotective, cytotoxic, antibacterial, and anti-inflammatory activities. PEC differs from other chromones because it possesses a C2-position phenylethyl substituent instead of a quite common phenyl group. This unique structural feature has provided neuroprotective activity within this chromone family that can be useful for the treatment of neurodegenerative disorders. They were obtained only by a few plant species such as *Eremophila georgei* (Myoporaceae), *Bothriochloa ischaemum* (Gramineae) (Wang et al., 2001), *Imperata cylindrica* (Gramineae), *Cucumis melo* L. (Cucurbitaceae), and *Aquilaria spp*. (Thymelaeaceae) (Ibrahim and Mohamed, 2015).

### C_2_-C_7_ Aldol Cyclization Derived Type III PKS Products

#### Stilbene Synthase—Stilbene, Resveratrol, and Pinosylvin

**Figure 5:**
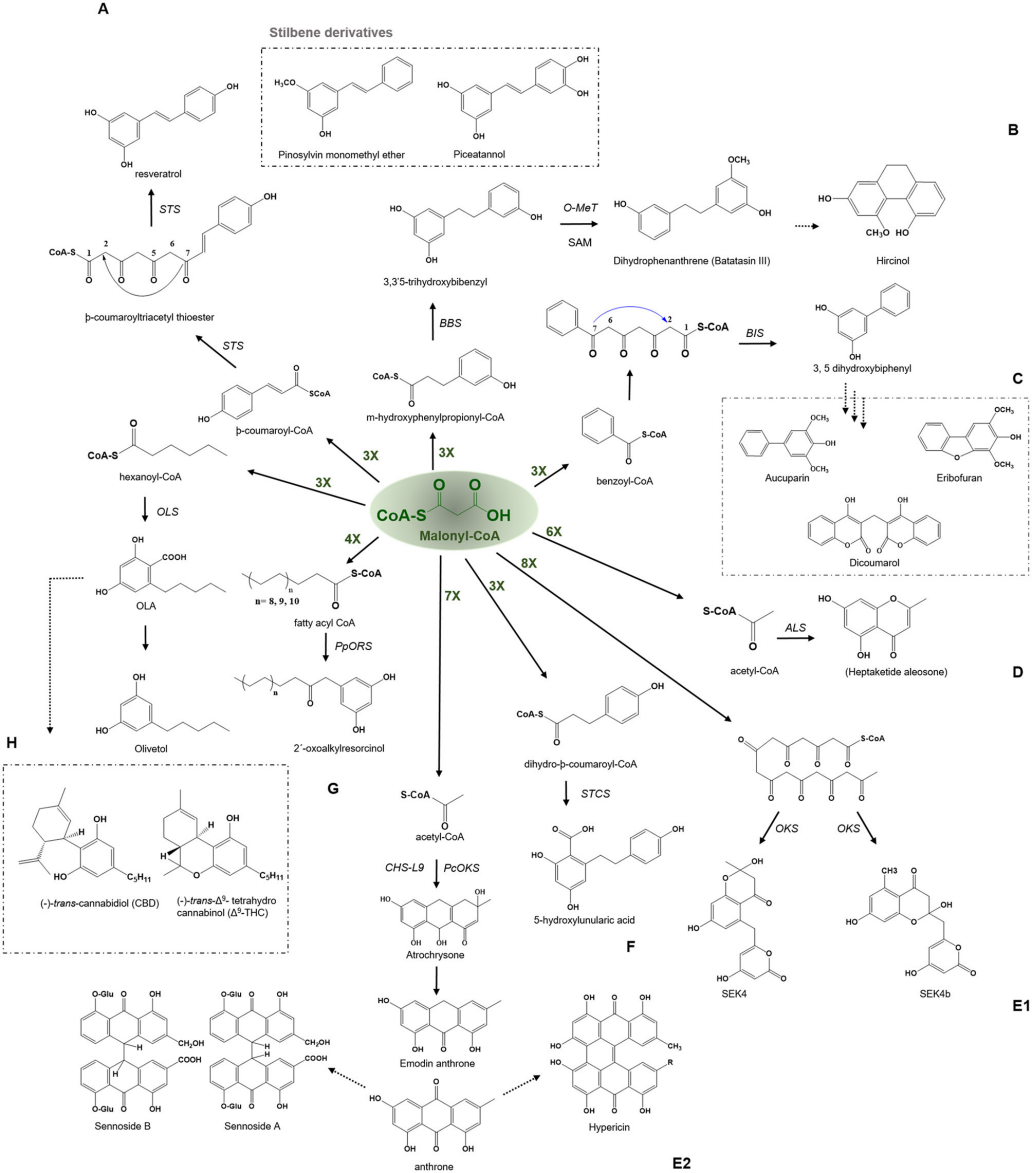
Examples of aldol type condensation reactions employed by various type III PKSs to produce a variety of polyketide products. Describes the reaction catalyzed by **(A)** Stilbene synthase (STS), **(B)** Bibenzyl synthase (BBS), **(C)** Biphenyl synthase (BPS), **(D)** Aleosone synthase (ALS), **(E1)** Octaketide synthase (OKS), **(E2)** Anthrone synthesizing CHS-L9 and PcOKS, **(F)** Stilbenecarboxylate Synthase (STCS), **(G)** 2′-Oxoalkylresorcinol Synthase (PpORS), and **(H)** Olivetol synthase (OLS). The dashed arrows represent the proposed pathways. The green-colored nX value represents the number of malonyl-CoA molecules employed in a reaction; O-Met, O-methyltransferase; SAM, S-adenosylmethionine.

Stilbene is biosynthesized by the stilbene synthase (STS) which shares 75–90% sequence identity with CHS and is thought to be evolved from gene duplication events. STS has a much-restricted occurrence and has been identified from *Vitaceae* (grapevine), *Gnetaceae, Dipterocarpaceae, Pinaceae* (pine), *Poaceae* (sorghum), *Fabaceae* (peanuts), *Leguminosae*, and *Cyperaceae* families (Austin et al., 2004). Stilbenes are simple polyphenolic compounds formed by similar iterative decarboxylative condensation of three molecules of malonyl-CoA with one molecule of -coumaroyl-CoA but follow a C_2_-C_7_ aldol type ring closure of the common tetraketide intermediate which produces a chemically different scaffold than CHS (Figure 5A). Additionally, CHS and STS are both capable of synthesizing products of the other cyclization type (STS produces chalcone and CHS produces stilbene in minor quantities) and display the potential to employ both types of cyclizations, preferring one to the other. The crystal structure of *Pinus sylvestris* STS reveals the basis of stilbene synthesis by showing the presence of cryptic thioesterase activity and an aldol switch. Stilbene and di- and tri-hydroxy stilbenes have accumulated considerable attention in recent times because of their diverse health benefits. In the last 12 years, almost 800 stilbenoids have been isolated from natural sources. The major group includes stilbene, stilbenoids, and stilbene oligomers. Stilbenoids can act as antimicrobial compounds, phytoalexins, and feeding deterrents, providing protection against pathogens and herbivores (Jeandet et al., 2010). In addition to their participation in defense mechanisms in plants, stilbenes, especially resveratrol (3, 5, 4′-trihydroxy-trans-stilbene) and its derivatives exhibit significant pharmacological properties and are thought to be of importance as antioxidants, cardioprotective, antitumor, and neuroprotective agents. Several efforts have been underway to understand the importance of resveratrol and to develop strategies for its over-production in heterologous host systems (Markus and Morris, 2008; Gambini et al., 2015). Different health benefits, including calorie restriction imitation, antioxidant, anti-inflammatory, antidiabetic, and anti-apoptotic properties, have made this an important molecule for drug development and is part of major clinical trials (Gambini et al., 2015; Berman et al., 2017; Thapa et al., 2019). The role of stilbenes as phytoalexins has been a subject of study for many researchers over the last three decades. The pinosylvin 3-*O*-methyl ether compound is the major active component that provides resistance to pines against nematodes and acts as a potent deterrent toward herbivores in several plant species. The antifungal nature of stilbene compounds make them a good candidate for wood decay prevention. In addition, certain constitutive stilbenes can also act as allelochemicals like piceatannol from *Carexspp*. which inhibits plant growth or plant photosynthesis, thus limiting the development of neighboring plants (Fiorentino et al., 2008).

#### Bibenzyl Synthase—Bibenzyls, Batatasin III, and Hircinol

The bibenzyl synthase (BBS) catalyzes the biosynthesis of 9,10-dihydrophenanthrene by the condensation of *m*-hydroxyphenylpropionyl-CoA with three molecules of malonyl-CoA to produce a 3, 3′5-trihydroxybibenzyl compound. The polyketide product is further mono-methylated by the action of an *S*-adenosyl methionine-dependent *O*-methyltransferase which upon subsequent oxidation transforms the bibenzyl compound into dihydrophenanthrenes, batatasins (Figure 5B). Batatasins are further metabolized to tricyclic phytoalexin 9, 10-dihydrophenanthrene derivatives such as hircinol, a compound known to accumulate in stressed or wounded orchid tissues. The expression of enzymes involved in the formation of dihydrophenanthrene has been observed to be 100-fold increased by the treatment with fungal elicitor *Botrytis cinerea*, suggesting the role of these metabolites as phytoalexins (Preisigmuller et al., 1995). The bibenzyl batatasins III from *Dioscorea* acts as a growth inhibitor for lettuce seed germination, lettuce hypocotyl elongation and wheat coleoptile elongation (Hashimoto and Tajima, 1978). Batatasin III has shown antidiabetic activity by inhibiting the inhibitor of α-glucosidase. Interestingly, there is a wide range of biological activities exhibited by phenanthrene derivatives, such as anti-inflammatory, anti-allergic, antimicrobial, cytotoxic, antifungal, phytotoxic, antifungal, antiplatelet aggregation, spasmolytic, antifibrotic, and inhibitory activities on nitrogen oxide (NO) production (Zhou et al., 2016).

#### Biphenyl Synthase—Biphenyl, Dibenzofurans, and Dicoumarol

Biphenyl and dibenzofurans are the phytoalexins found in the Rosaceae plant family. The chemical core of these compounds is biosynthesized by the biphenyl synthase, BPS, which shows a preference for the benzoic acid derived starter molecules similar to BPS and performs a three-round of condensation reaction with malonyl-CoA to yield a tetraketide intermediate (Figure 5C) (Liu et al., 2007). The intermediate undergoes intramolecular aldol condensation to produce 3, 5-dihydroxybiphenyl, a precursor molecule for two major classes of plant phytoalexins, e.g., dibenzofuran and biphenyl aucuparin. BIS shares almost 60% amino acid sequence identity with other members of the CHS superfamily of enzymes and group near BPS in an evolutionary tree due to its specific requirements for benzoic acid derivatives [Figure 4 (10) and (11)]. The expression of biphenyl and dibenzofurans was found to transiently and rapidly increase in response to the treatment with yeast extract in a cell suspension of *Sorbus aucuparia*. Furthermore, various elicitation studies with different elicitors have shown the expression of one of these phytoalexins, where aucuparin expression was induced by yeast extracts, whereas eriobofuran as the main inducible constituent was accumulated by the fire blight bacterium, *Erwinia amylovora*, and scab causing fungus, *Venturia inaequalis* (Hüttner et al., 2010). The medicinally important 4-hydroxycoumarin derivative, dicoumarol (anticoagulant) is also synthesized by the action of BIS by condensing one molecule of salicoyl-coA with one unit of malonyl-CoA. The crystal structure of BIS from *Malus domestica* has shown insights into the functional diversification of this enzyme, brought by mutations in the active site cavity which limit the preferences of its starter substrates and render it incompetent for the use of larger -coumaroyl-CoA molecules (Beerhues and Liu, 2009; Stewart et al., 2017).

#### Aleosone Synthase—Chromones, Aloesones, and Aloesin

The aleosone synthase (ALS) is a key enzyme in the biosynthesis of heptaketide chromone aloesone derivatives (Figure 5D) (Abe et al., 2004). ALS shares ~60% sequence identity with CHS and retains catalytic residues and the overall fold. The cavity of ALS is almost equivalent to that of the CHS; however, its cavity is substantially bigger than other pyrones synthesizing enzymes such as 2-PS. On the contrary, the cavity volume of 2- PS is 1/3rd of the CHS due to a steric bulk at the active site residues positions (197 and 338) obstructing the coumaroyl-binding pocket, thus only allowing a smaller starter unit. Although product profiles and elongation reactions differ between ALS and 2-PS, similar cavity residue positions (197, 257, and 338 of CHS) are affected in both ALS and 2-PS. In ALS, Thr197, Gly256, and Ser338, the active site residues lining the initiation/elongation cavity (of CHS), are uniquely replaced with Ala, Leu, and Thr, respectively (Figure 2). Gly256 residue is crucial for determining starter substrate selectivity, while Thr197 controls the polyketide chain length whereas the Ser338 directs the linear polyketide intermediate to extend into the enzyme's pocket (Abe et al., 2006). Despite its structural similarity to CHS, the recombinant ALS was unable to accept natural starters, such as -coumaroyl-CoA or other aromatic CoA esters (cinnamoyl-CoA and benzoyl-CoA), instead effectively utilized acetyl-CoA as a starter and condensed with six molecules of malonyl-CoA to generate an aromatic heptaketide, aloesone *in-vitro* reaction. ALS from rhubarb (*R. palmatum*) and aloe (*A. arborescens*) catalyzes the condensation of acetyl-CoA with six molecules of malonyl-CoA followed by an aldol condensation to generate a heptaketide aromatic pyrone 6-(2-(2,4-dihydroxy-6-methyl phenyl)-2oxoethyl)-4-hydroxy-2-pyronechromone with trace amounts of hexaketide pyrones 6-(2,4-dihydroxy-6-methyl phenyl) 4-hydroxy-2-pyrone and octaketide pyrones SEK4 and SEK4b. Finally, the unstable heptaketide pyrone is subsequently spontaneously isomerized to β-ketoacid chromone, followed by decarboxylation to produce aloesone heptaketides. Aloesone O-glucoside (7-O-β-D-glucopyranoside) from rhubarb and aloesone C-glucoside (8-C-β-D-glucopyranoside) (aloesin) from aloe (*Aloe ferox*, Liliaceae) has anti-inflammatory effects (Radha and Laxmipriya, 2015).

#### Octaketide Synthase—Anthraquinone and Emodin Anthrone

Anthraquinone is an anthracene derivative in plants and is the precursor of emodin anthrone that could further act as a precursor of hypericin found in the *Hypericum perforatum* L. St John's wort. Hypericin has antidepressant activities and a role in insect-plant protection. The photosensitizer activities of hypericin makes it a potential antitumor and antiviral agent (Karppinen et al., 2008). The two anthraquinone glycosides from *Cassia angustifolia* Vahl, known as Sennoside A and B, have laxative effects as well as having notable anticancer activity in multiple cell lines (Chetri et al., 2016). The anthranoid scaffold of anthraquinone is synthesized by an octaketide synthase (OKS) that utilizes acetyl-CoA as a starter substrate and condenses with seven units of malonyl-CoA to yield a linear octaketide intermediate (Figure 5E1). The incorrect folding of the intermediate could result in shunt products, SEK4 and SEK4b (Abdel-Rahman et al., 2013). Abdel-Rahman et al. (2013) showed the generation of torosachrysone (tetrahydroanthracene) and emodin anthrone in the *in vitro* reaction using acetyl-CoA and [2^−14^C] malonyl-CoA with yeast extract-treated cell cultures. Kang et al. (2020), have recently identified a PKS gene, CHS-L9, responsible for the biosynthesis of atrochrysone carboxylic acid and endrocrocin anthrone in the medicinal plant *Senna tora*, by utilizing a genome-based screening coupled with biochemical characterization (Figure 5E2). The study is the first to identify a role of Type III PKS in the synthesis of anthraquinone, however, the study did not report the detection of the final product, emodin, or other fully oxidized products. While it has previously been shown, HpPKS2, from *H. perforatum* holds the potential to synthesize octaketides (SEK4 and SEK4b) *in vitro*, and the expression of this gene is corroborated with the location of hypericin accumulation in the dark glands of leaves and flower buds in *H. perforatum* (Karppinen et al., 2008). The above studies show the participation of Type III PKSs in the biosynthesis of plant anthraquinones and derivatives.

Anthraquinones derivatives have been identified from a variety of species such as *Rheum, Rumex, Aloe*, and *Cassia* containing medicinal and industrial properties. Most anthraquinones are laxative and emodin has been linked to a variety of biological activities including, anti-inflammatory, antimicrobial, anticancer, antidiabetic, DNA-binding, and vasorelaxant (Chien et al., 2015). For a detailed description of the bioactivities of anthraquinones and derivatives, interested readers can refer to the following articles (Dave and Ledwani, 2012; Chien et al., 2015; Su et al., 2020).

#### Stilbene Carboxylate Synthase-5-Hydroxylunularic Acid

The stilbene carboxylate synthase (STCS), from *Hydrangea macrophylla*, catalyzes the typical reaction of the stilbene synthase except it retains the terminal carboxyl group to produce a 5-hydroxylunularic acid through condensation of dihydro--coumaroyl-CoA substrate with three molecules of malonyl-CoA (Figure 5F) (Eckermann et al., 2003). Worth noting that with -coumaroyl-CoA as a starter, STCS produces only bisnoryagenin and coumaroyl triacetic acid lactone (CTAL). Despite the mechanistic similarity between STS and STCS, recent structural studies have shown that STCS is closer to CHS. CTAS and STCS from the Hydrangea varieties are now considered to be identical in their function because the enzymes are similar except for five amino acid replacements, CTAS and STCS grouped in a separate clade distinct from STS [Figure 4 (24) and (25)]. Functionally, lunularic acid and its analogs have growth-inhibiting activity and inhibit germination in liverwort. Lunularic acid shares remarkable biological and structural similarities to abscisic acid (ABA) which explains its ABA-like activity (Yoshikawa et al., 2002).

#### 2′-oxoalkylresorcinol Synthase—Oxoalkylresorcinol, Benzoquinone, and Sorgoleone

The 2′-oxoalkylresorcinol synthase is involved in the synthesis of 2′-oxoalkylresorcinol in the bryophyte *Physcomitrella patens* and has recently been cloned and characterized to understand the evolution of land plants due to its high possibility to be the most common ancestor of the Type III PKSs of the land plants. The *in planta* function of PpORS is most likely to prevent dehydration in the moss as the recent study has shown defects in cuticular structure and increased dehydration susceptibility, with increased color permeability in case of the *PpORS* mutant (Li et al., 2018). PpORS showed the ability to condense a very-long-chain fatty acyl-CoA with four molecules of malonyl-CoA to catalyze decarboxylative aldol cyclization to yield a pentaketide 2′-oxoalkylresorcinol (Figure 5G). However, PpORS failed to produce alkylresorcinol molecules solely, emphasizing the crucial role of the oxo group in the final product in a cuticle, where the pathway genes are expressed.

Mutagenesis studies have revealed the importance of Ala286 in determining the starter specificities since the A286F mutant failed to produce pentaketide 2′-oxoalkylresorcinol and only produced triketide alkylpyrones from fatty acyl-CoA substrates with shorter chains (Kim et al., 2013). Alkylresorcinol belongs to a group of phenolic secondary metabolites and has been reported by many different species of higher plants. They play a major role in plants as molecules of defense, allelochemicals, and phytoanticipins (Baerson et al., 2010). These alkylresorcinols and derivatives are mainly confined within seed coats of wheat, rye, barley, and other cereals. The lipid benzoquinone sorgoleone (alkylresorcinol derivative) produced from *Sorghum bicolor* is the classic example of allelochemical with antifungal activities and current reports suggest its polyketide mode of origin. Because of their amphipathic nature, alkylresorcinols and derivatives form thin exudate layers, completely covering root systems, thus providing a continuous defensive boundary. Alkylresorcinol producing two Type III PKSs (designated ARS1 and ARS2) are identified in hair root cells of *S. bicolor*, with a possible role in sorgoleone production (Cook et al., 2010). Similar efforts are being carried out in other cereals such as rice (*Oryza sativa*) to identify alkylresorcinol synthesizing genes involved in the defense mechanisms. Recent studies have demonstrated a growth inhibitory effect of whole-grain derived alkylresorcinols in different cancer cell lines such as colon, breast, lung, CNS, hepatocarcinoma, and ovarian. Alkylresorcinols and derivatives can be interesting candidates for designing therapies for cancer prevention (Kruk et al., 2017).

#### Olivetol Synthase—Olivetol and Cannabinoids

The olivetol synthase (OLS) is associated with the synthesis of olivetol in *Cannabis sativa* and is expressed in flowers and rapidly expanding leaves which are the source of cannabinoids. OLS catalyzes the decarboxylative condensation of hexanoyl-CoA starter molecule with three molecules of malonyl-CoA followed by an aldol cyclization to generate olivetol with tetra- and tri-ketide pyrones (Figure 5H) (Taura et al., 2009). OLS shares ~65% sequence identity with CHS and retains catalytic residues at the corresponding sites. OLS is functionally similar to plant STS, however, with the restricted starter specificity as it does not incorporate the coumaroyl-CoA starters and prefers C_4_-C_8_ aliphatic acyl-CoAs, possibly due to the presence of Ala, Met, Leu residue sites at Thr132, Thr194, and Thr197 in CHS which have defining role in starter specificity (Figure 2). Interestingly, OLS shows a remarkable ability to utilize NAC tethered synthetic starters.

*C. sativa* is the only known producer of cannabinoids. Cannabinoids consists of alkylresorcinol and monoterpene groups and their alkylresorcinol moieties are derived from olivetolic acid (OLA), an intermediate in the olivetol biosynthesis. The catalytic activity and expression profile of OLS highlight the possibility of OLA formation *via* the OLS pathway (Taura et al., 2009). It was worth noting that the same study did not show the direct link between the OLS expression and olivetolic acid formation, thereby the *in planta* function of OLS remains elusive.

Cannabinoids have a variety of pharmaceutical and health benefits and are in high demand for pharmaceutical and medicinal purposes. The two major components of cannabinoids; Cannabidiol (CBD) and Tetrahydrocannabinol (THC) are linked with health-benefiting activities. Although natural and synthetic cannabinoids and derivatives possess different health and medicinal benefits, their use remain illegal due to the serious risk of drug abuse and related negative effects. Its use has been studied in treating various health conditions like pain, inflammation, multiple sclerosis (MSS), anorexia, stroke, PTSD, neurodegenerative disorders (Parkinson's disease, Huntington's disease, Tourette's syndrome, and Alzheimer's disease), epilepsy, glaucoma, osteoporosis, schizophrenia, cardiovascular disorders, cancer, obesity, and metabolic syndrome-related disorders (Kogan and Mechoulam, 2007).

The current status of cannabis-derived compounds approved by the FDA permits usage of dronabinol (Synthetic Δ9-THC, Marinol®) and nabilone (a synthetic analog of Δ9-THC, Cesamet®) for counteracting the symptoms of nausea and vomiting associated with chemotherapy and to stimulate appetite in AIDS patients. Two more drugs based on cannabinoids; Nabiximols (Sativex®), a 1:1 ratio of Δ9-THC: CBD indicated in the symptomatic relief of multiple sclerosis and as an adjunctive analgesic treatment in cancer patients and the second is the investigational drug Epidiolex®, a concentrated CBD oil (>98% CBD), which is an anti-seizure medication for Dravet and Lennox-Gastaut syndromes (National Academies of Sciences, 2017).

### C_5_-O-C_1_ Lactonization Derived Type III PKS Products

#### Coumaroyl Triacetic Acid Synthase—Coumaroyl Triacetic Lactone and Hydramacrosides B

**Figure 6:**
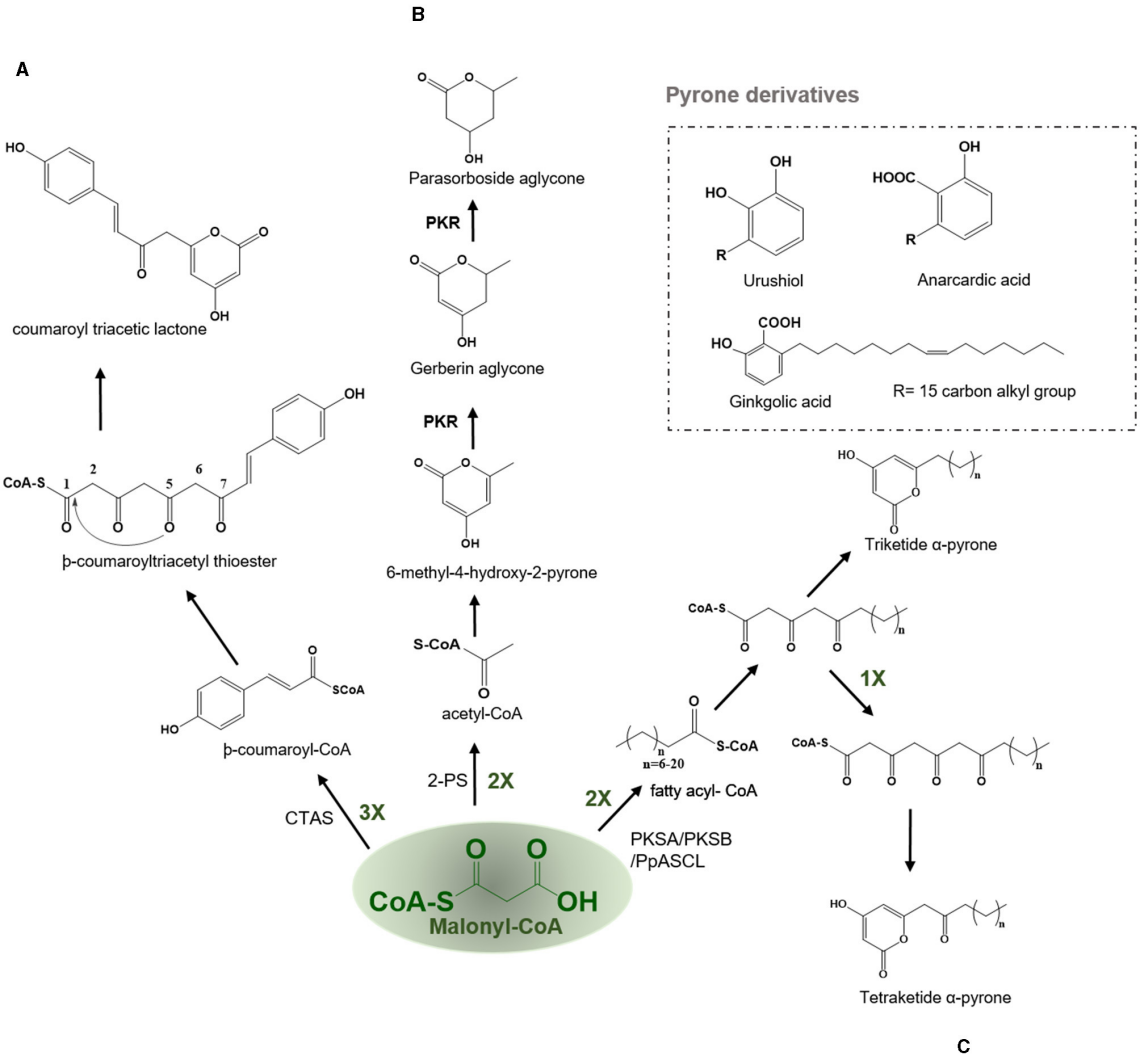
Examples of lactonization type reaction employed by various type III PKSs to produce a variety of polyketide products. Describes the reaction catalyzed by **(A)** Coumaroyl triacetic acid synthase (CTAS), **(B)** Pyrone synthase (2-PS), and **(C)** PKSA (lap6) and PKSB (lap5); PKS, polyketide reductase; PpASCL, anther specific chalcone like synthase.

In the CHS catalyzed reaction, the common tetraketide intermediate can undergo cyclization through lactonization type ring closure by the action of the coumaroyl triacetic acid synthase (CTAS) to yield a coumaroyl triacetic lactone (CTAL) (Figure 6A). In nature, lactonization can proceed non-enzymatically yielding triketide and tetraketide pyrones which are often the derailment products of the main Type III PKS reaction. The coumaroyl triacetic acid lactone in *Hydrangea macrophylla var. thunbergii* and dihydroxymethylphenyl methylpyrone compounds are thought to be premature hydrolysis products catalyzed by the CTAS. Therefore, it has been argued that CTAL is a common by-product for all CHS and STSs, but its production was limited to *in vitro* reactions (Akiyama et al., 1999a). HmS from *Hydrangea macrophylla var. hunbergia* produces CTAL as the major product *in vitro* and is responsible for the biosynthesis of hydramacroside B in the mother plant (Akiyama et al., 1999a,b). Hydramacrosides B have shown to exert an inhibitory effect on the histamine release from the rat mast cells induced by the antigen-antibody reaction (Matsuda et al., 1999).

#### Pyrone Synthase—Pyrones, Gerberin, and Parasorboside

2-PS catalyzes the synthesis of a triacetic acid lactone (TAL) from the condensation of an acetyl-CoA with two molecules of malonyl-CoA *via* a triketide intermediate using lactonization based cyclization (Figure 6B). Pyrones in plants are only made by a few species, such as the *Gerbera hybrida* (Asteraceae) which is known to produce gerberin and parasorbosides, with a role in insect and pathogen resistance. In addition, bis-noryangonin (triketide pyrone) produced by the lactonization reaction of -coumaroyltriacetyl thioester in kava (*Piper methysticum*) is an effective anti-anxiety compound (Dewick, 2002). The basic reaction scheme followed by a 2-PS is to condense a starter acetyl-CoA with two molecules of elongator malonyl-CoA to produce a triketide intermediate. The pyrone synthase (PS) catalyzes the production and subsequent cyclization of a common triketide into a 6-methyl-4-hydroxyl-2-pyrone (Helariutta et al., 1995). One exception to this rule is that long-chain fatty acyl CoA esters such as palmitoleoyl (C_16_)–CoA can act as starter substrate for malonyl-CoA chain extension which results in the formation of alkyl polyphenols such as urushiol and ginkgolic acid (anacardic acid), the allergic substances of lacquer tree (*Rhus verniciflua*, Anacardiaceae) and ginkgo tree (*Ginkgo biloba*, Ginkgoaceae), respectively (Dewick, 2002). The *in planta* functions of these anacardic acids are identified to be as physical trap and anti-pests agents. The second class of pyrones is dibenzo-α-pyrones which are an important group of heptaketide coumarin derivatives, with a fused tricyclic nucleus, that are known as dibenzo-α-pyranones, 6H-benzo[c]chromen-6-ones, and 6H-dibenzo [b, d] pyran-6-ones. They are synthesized by various microorganisms and also produced by metabolization of plant-derived ellagitannins and ellagic acid by the intestinal bacteria. They are usually isolated from various species of plants, fungi, microorganisms, and animals. Most of these dibenzo-α-pyrones have an extensive range of biological activities, including toxicity to humans and animals, cytotoxicity, phytotoxicity, antioxidant, anti-allergic, antimicrobial, and anti- acetylcholinesterase activities. In addition, the dibenzo-α-pyrones are key intermediates in the synthesis of cannabinoids and other pharmaceutically important compounds such as progesterone, androgen, glucocorticoid receptor agonists as well as endothelial proliferation inhibitors and antidyslipidemic agents (Mao et al., 2014).

#### PKSA (Lap6) and PKSB (Lap5)-Alkylpyrones and Sporopollenin

Th polyketide synthase A (PKSA) (anther specific chalcone-like synthase) and PKSB from *Arabidopsis thaliana* are recently found to be involved in the synthesis of alkylpyrones and hydroxyalkylpyrones by utilizing the starter molecules synthesized by acyl-CoA synthetase5 (ACOS5). The expression of both type III PKSs and ACOS5 have been closely regulated and the role of ACOS5 in the formation of sporopollenin has already been established. PKSA and PKSB are specifically and transiently expressed in tapetal cells during microspore development in *Arabidopsis* anthers. Sporopollenin is the main constituent of the pollen exine and is chemically the most robust structure known from the pollen cell wall. Recently, the importance of PKSA and PKSB has been established in the generation of hydroxylated α-pyrones, possible precursors for sporopollenin formation. Mutants compromised in the expression of these PKS genes displayed pollen exine layer defects and the double mutants were completely exine deficient and sterile (Kim et al., 2010). PKSA and PKSB catalyze the decarboxylative condensation of malonyl-CoA with medium-chain to long-chain, and hydroxylated fatty acyl-CoA to yield tetraketide α-pyrones which are required for sporopollenin formation during the pollen grain development (Figure 6C) (Kim et al., 2010). PpASCL, an anther-specific chalcone-like synthase in moss *Physcomitrella patens* is an ortholog of PKSA, that is involved in the generation of alkyl and hydroxyalkyl α-pyrones by utilizing saturated acyl-CoAs (C_6_-C_20_), unsaturated acyl-CoAs (C16:1 or C18:1), or hydroxyl fatty acyl-CoAs. These hydroxylated pyrones provide the building blocks for the synthesis of sporopollenin in the moss spore cell wall (Colpitts et al., 2011). Notably, both PKSA and PpASCL can utilize -coumaroyl-CoA to yield bisnoryangonin. The ability of these enzymes to accommodate large starter substrates is due to the presence of a smaller Gly205 in PKSA and Gly225 in PpASCL, at the place of a bulkier Thr197 residue in alfalfa CHS (Figure 2). This change expands the entrance of the acyl-binding tunnel of these enzymes (Colpitts et al., 2011).

### Non-cyclized Based Type III PKS Derived Products

#### Benzalacetone Synthases—Benzalacetone, Quinolone Alkaloids, and Phenylbutanoids

**Figure 7:**
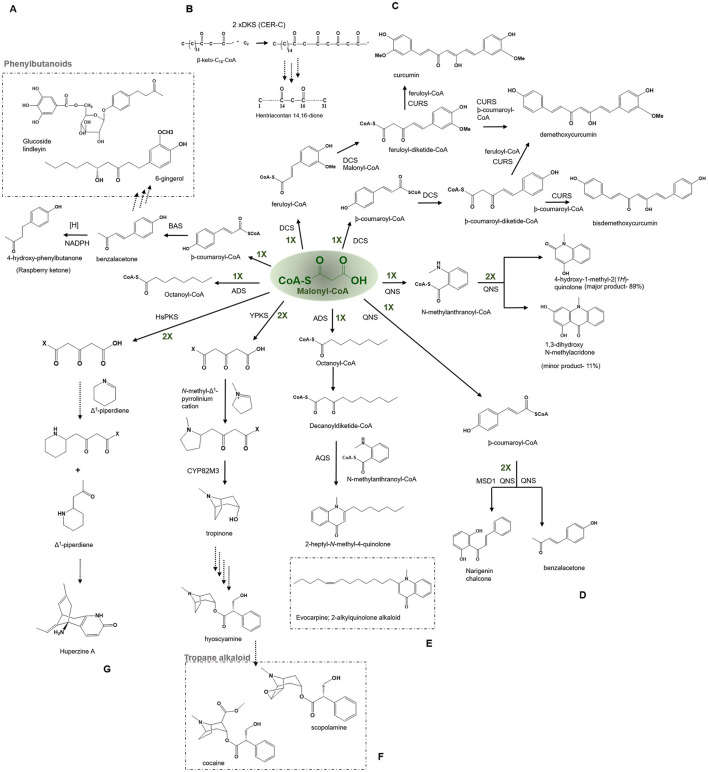
Examples of non-cyclization based -type III PKSs to produce a variety of polyketide products. Describes the reaction catalyzed by **(A)** Benzalacetone synthases (BAS), **(B)** β-Diketone Synthase (DKS), **(C)** Diketide- CoA synthase (DCS) and Curcumin synthase (CURS1), **(D)** Quinolone synthase (QNS), **(E)** Alkyldiketide-CoA synthase (ADS) and alkylquinolone synthase (AQS), **(F)** polyketide like synthase (YPKS), and **(G)** Polyketide synthase (HsPKS); CYP82M3, cytochrome oxidase.

The benzalacetone synthase (BAS) has a high (~70%) sequence identity with CHS, but still catalyzes a different reaction from the typical CHS type mechanism. It catalyzes a one-step decarboxylative condensation of -coumaroyl-CoA with a single molecule of malonyl-CoA to produce a diketide benzalacetone that upon reduction produces 4-hydroxy-phenylbutanone (pHPB) (Figure 7A) (Abe et al., 2003). The BAS fold is identical to that of the CHS, with comparable cavity volumes, but the gatekeeper Phe215 corresponding to the CHS residue position is mutated in BAS, allowing for early termination at the diketide stage, which upon subsequent decarboxylation by the benzalacetone reductase forms a benzalacetone (Borejsza-Wysocki and Hrazdina, 1996; Abe et al., 2003). The Leu208 in BAS (at the gatekeeper Phe215 position) might be a driver for the biosynthesis of benzalacetone in *Rheum Palmatum*. This substitution may hinder the subsequent chain extension of the diketide intermediate. In addition, the enzyme utilizes an alternate coumaroyl-binding pocket to accommodate the starter-CoA as the original entrance of the CHS is obstructed sterically in BAS by Leu125, Leu208, and Ser331. The conventional coumaroyl-binding pocket of CHS is restored in the I207L/L208F mutant of BAS, thus allowing the mutant to catalyze CHS-like reactions. The comparison of crystal structures of wild-type BAS and I207L/L208F mutant has revealed insights regarding the novel catalytic mechanism employed by BAS which proceeds *via* thioester bond cleavage of the enzyme-bound diketide intermediate and the final decarboxylation reaction to produce benzalacetone (Abe et al., 2001; Morita et al., 2010). BAS has been cloned and characterized from *R. palmatum* and *Rubus idaeus*; however, the BAS from *R. idaeus* is bifunctional and capable of synthesizing both benzalacetone and naringenin chalcones. BAS from *R. palmatum* can utilize malonyl or methylmalonyl-CoA and additionally accepts bulkier starter units such as *N*-methylanthraniloyl-CoA (or anthraniloyl-CoA) to produce 4-hydroxy-2(1H)-quinolones, precursors of quinolone alkaloids occurring in abundance in plants from the Rutaceae family but not found in rhubarb. Phenylbutanoids, a biologically important class of natural products from the Zingiberaceae family, receive the C_6_-C_4_ moiety by the BAS catalyzed pathway. Phenylbutanoids possess various pharmacological activities including anti-inflammatory, antioxidant, and hypolipidemic (Sinha et al., 2005). One of the important anti-inflammatory phenylbutanoids synthesized by BAS is glucoside lindleyin in the medicinal plant rhubarb (*Rheum palmatum*) (Figure 7A) (Mander and Liu, 2010). Benzalacetone, also known as raspberry ketone, is the characteristic aroma compound of raspberries. The toxicity of the pHPB to phytopathogenic fungi and rapid induction of the pathway enzymes suggest the possibility of this being employed in plant defense response. Raspberry ketone has various industrial applications such as in flavoring, aroma, fragrances, dietary supplements to aid weight loss, and male-specific lures used in agriculture (Vargas et al., 2010; Lee, 2016).

#### β-Diketone Synthase—β-Diketone and Wax Polyketides

The *cer c*, β-diketone synthase (DKS) belonging to the *Cer-cqu* gene cluster identified in barley is involved in the synthesis of β-diketones forming long, thin crystalline tubes and are components of the epicuticular wax layer present on the aerial surfaces of the plant (von Wettstein-Knowles, 2017). The cluster comprises *cer c* (DKS), *cer u* (P450 hydroxylase), and *cer q* (lipase/carboxyl transferase) genes tightly linked for participating in the biosynthetic pathway of β-diketones, hydroxy-β-diketones, and esterified alkan-2-ols. The β-diketones component of the epicuticular wax is found in Eucalyptus, Acacua, Dianthus, Festuca, Buxus, Rhododendron, and Hosta lancufolia. The β-diketones are comprised of primarily 29, 31, and 33 carbon skeletons. DKS was shown to utilize shorter fatty acyl-CoAs (C_12_-C_16_) but not C_18_ *in vitro* reaction and the 3-oxo-C_16_-CoA was identified to be the most preferred starter for this enzyme while 3-oxo-C_18_-CoA was the precursor of choice *in vivo* (Mikkelsen, 1984). DKS performs two rounds of extension of the 3-oxo-C_16_-CoA starter to yield a tetraketide intermediate in barley, which is typical of the other type III PKSs. The intermediate could further be elongated by an FAE (fatty acyl elongase) complex to reveal a β-diketone carbon skeleton. However, it is currently not clear whether FAE produces the six elongations or DKS itself to produce the final products (Schneider et al., 2016) (Figure 7B).

These epicuticular waxes in plants provide a water barrier, protect shoots from desiccation and serve as the first line of defense against pathogens and herbivores, in addition to reflecting harmful UV radiation. The role of the type III PKS system in the synthesis of epicuticular waxes further expands the chemical potential of these enzyme systems. This study further enhances our understanding of the *in planta* significance of type III PKSs that could be exploited for commercial purposes.

#### Diketide-CoA Synthase and Curcumin Synthase—Curcumin, Demethoxycurcumin, Bisdemethoxycurcumin

The diketide-CoA synthase (DCS) in conjunction with curcumin synthase 1 (CURS1) participates in the biosynthesis of curcuminoids found within the dried rhizome of the perennial herb *Curcuma longa Linn* (Turmeric). Chemically curcuminoids or curcumin are polyphenols that are abundantly present in the spice turmeric (Haldi). They are a mixture of curcumin, demethoxycurcumin, and bisdemethoxycurcumin. The proposed C6–C7–C6 curcuminoid scaffold is synthesized by employing a three-step reaction from the phenylpropanoids in which the first malonyl-CoA condenses with feruloyl-CoA, to produce a feruloyldiketide-CoA by the action of DCS, subsequently the diketide gets converted into a β-keto acid through the hydrolysis followed by the second condensation with another molecule of feruloyl-CoA to produce curcumin by the action of CURS1 (Figure 7C) (Katsuyama et al., 2009) (Abe and Morita, 2010). Both DCS and CURS1 can use other starters such as coumaroyl-CoA and malonyl-CoA; however, feruloyl-CoA is preferred, the enzymes share 63% sequence identity [Figure 4 (7) and (8)]. Type III PKSs, CURS2, and CURS3 have also been cloned and characterized from *Curcuma Longa Linn* which shows starter specificity for feruloyl-CoA as well as coumaroyl-CoA. The presence of three different type III PKSs in turmeric might be responsible for the formation of a different mixture of curcuminoids. The structural analysis of CURS1 revealed the presence of an unusual hydrophobic pocket in the CoA binding tunnel, which was created because of the different orientations of the gatekeeper Phe265 alongside the substitution of active Ser338 site with Gln338 that suggests the requirement of the hydrophobic cavity in allowing hydrophobic interaction between CURS1 and β-keto acid (Katsuyama et al., 2011). A new curcumin synthase (ZoCURS) has recently been identified from ginger (*Zingiber officinale*) This curcumin synthase has a different starter preference and accepts 3-(4-hydroxyphenyl) propionyl-CoA to produce tetrahydrobisdemethoxycurcumin and similar products (Zhang et al., 2016).

Curcuminoids have various pharmacological activities that make them an interesting topic for research. Curcumin (dipheruloylquinone) is the most explored of the so-called curcuminoids, a family of chemopreventives. Recently, different health properties of curcuminoids have become very interesting, including strong antioxidant properties, inhibitory effects on COX-2, LOX and NFB, anti-cancer, anti-angiogenic, neuro-protective, wound healing, antidiabetic activities, and role in epigenetic regulation mechanisms (Maheshwari et al., 2006; Bengmark et al., 2009; Amalraj et al., 2017).

#### Quinolone Synthase—Quinolone Alkaloids

Quinolone alkaloids are anthranilic acid-derived alkaloids present mainly in the Rutaceae plant family. The quinolone synthase (QNS) involved in the synthesis of quinolone alkaloid is cloned from *Aegle marmelos* (bael). It catalyzes the condensation of starter *N*-methylanthraniloyl-CoA with three molecules of malonyl-CoA which spontaneously cyclize to 4-hydroxy-2(1H)-quinolone (major product, 89%) and acridone scaffold (minor product, 11%) (Figure 7D) (Resmi et al., 2013). QNS is interesting in the context that it shows promiscuous starter specificity and can utilize both smaller acyl-CoAs and bulkier *N*-methylanthraniloyl-CoA substrates *in vitro* reaction, an enzymatic potential shown by the ACS and an F215S mutant of msCHS. Mutagenesis studies of a double mutant, named MSD1 (S132T and A133S) showed a change in the active site cavity that imparts it a unique ability to utilize bulkier starter molecules and completely transformed QNS to CHS (Resmi et al., 2013). These naturally occurring quinolones have potent medicinal properties and also give impetus to the design of synthetic quinolones as antimalarials drug targets (Bawa et al., 2010). Among various properties of quinolone alkaloids, the most prominent ones are antimalarial (quinine, chloroquine, mefloquine, and amodiaquine), motor inhibitory (Skimmianine) (Cheng, 1986), anti-platelet aggregation (Chen et al., 2000), and cytotoxicity against HeLa cell line (Jansen et al., 2006).

#### Alkylquinolone Synthase—Evocarpine and 2-Alkyquilonone Alkaloids

Some Type III PKSs catalyze the condensation reactions with CoA thioesters to produce polyketide moieties with R_1_-C-R_2_ scaffolds (Abe, 2020). Two functionally distinct Type III PKSs, namely, alkyldiketide-CoA synthase (ADS) and alkylquinolone synthase (AQS), participate in the biosynthesis of evocarpine, a 2-alkylquinolone alkaloid produced in *Evodia rutaecarpa* (Matsui et al., 2017; Abe, 2020). The enzymes share a 61% sequence identity and ADS initiates the reaction by performing a decarboxylative condensation of fatty acyl-CoA (C8-C12) with a malonyl-CoA extender to generate an alkyldiketide-CoA. Consecutively, AQS catalyzes the combination of the starter *N*-methylanthraniloyl-CoA with the diketide acid (formed by a non-enzymatic hydrolysis of alkyldiketide-CoA intermediate) through C-C and the C-N bond formations to generate 2 AQ scaffolds (Figure 7E) (Matsui et al., 2017). The biosynthesis of 2 AQ in *E. rutaecarpa* resembles the curcumin biosynthesis in turmeric described above. The X-ray crystal structure analysis and site-directed mutagenesis studies of ADS and AQS reveal a unique geometry of the active site and a new binding CoA tunnel architecture governing ADS and AQS substrate and product specificities. 2-AQ displays a broad range of pharmaceutical and biological properties such as antibacterial, cytotoxic, anticholinesterase and quorum sensing activities (Wang et al., 2013).

#### Tropane Alkaloid Synthase-Tropane Alkaloids

Tropane alkaloids (TA) are pharmaceutically significant plant secondary metabolites with a characteristic 8-azabicyclo [3.2.1] octane core bicyclic structure and are abundantly present in Solanaceae and Erythroxylaceae; examples include hyoscyamine and scopolamine, and cocaine and calystegines (Huang et al., 2019, 2021; Kohnen-Johannsen and Kayser, 2019). AbYPKS is the atypical Type III PKS from *Atropa belladonna* involved in the tropinone biosynthesis, the first intermediate in tropane alkaloid biosynthesis. AbYPKS catalyzes decarboxylative condensation of an unconjugated *N*-methyl-Δ^1^-pyrrolinium cation starter with two molecules of malonyl-CoA extender to generate a 4-(1-methyl-2-pyrrolidinyl)-3-oxobutanoic acid. Additionally, *A*bCYP82M3 acts upon the 4-(1-methyl-2-pyrrolidinyl)-3-oxobutanoic acid to produce tropinone (Figure 7F) (Bedewitz et al., 2018). Furthermore, the *in vitro* assay of AbYPKS with substrates *N*-methyl-Δ^1^-pyrrolinium cation and malonyl-CoA resulted in the production of pyrrolidine alkaloids; hygrine and cusohygrine. Recently, two atypical Types III PKS, named EcPYKS1 and EcPYKS2 have been identified in *Erythroxylum coca* that is involved in the biogenesis of coca alkaloid. The current data suggests these enzymes utilize malonyl-CoA as a sole substrate to produce 3-oxoglutaric acid and belong to a unique non-CHS class of Type III PKSs. The structural analysis of AbYPKS and EcPYKS1 revealed differences in the residues (Arg134Thr and Ser340Gly in EcPYKS1) responsible for the stabilization of the intermediate (Kim, 2020; Lichman, 2021). These recent studies highlight the role of atypical Type III PKSs in catalyzing the second ring closure of the bicyclic ring leading to the production of tropane alkaloids.

Scopolamine has a variety of medicinal properties, it is marketed to treat nausea, vomiting, motion sickness, and spasms due to its anticholinergic effect (Ullrich et al., 2017). Cocaine, on the other hand, is an illicit psychoactive drug and is the only naturally occurring local anesthetic (Sayhan et al., 2017).

#### Lycopodium Alkaloids

Lycopodium alkaloids (LA) are classified as nitrogen-containing heterocyclic metabolites with diverse and stereochemically complex structures that have garnered attention due to their potent biological activities (Lichman, 2021). Some LA, such as huperzine A (HupA), acts as an inhibitor of acetylcholinesterase thereby are promising candidates for the treatment of Alzheimer's and myasthenia gravis disease in addition to harboring cytotoxic and neuroprotective activities (Ma and Gang, 2004; Wang et al., 2020). Classically, LAs have been categorized into four major groups (lycopodine, lycodine, fawcettimine, and phlegmarine) and since the discovery of the first LAs, lycopodine, around 400 LAs have been identified from the *Lycopodiaceae* and *Huperziaceae* families. Interested readers can consult review of Ma and Gang (2004) about these compounds for a detailed description.

The biosynthesis mechanism of LA remained poorly understood in plants until now. In a recent report by Wang et al. (2020), on the biosynthesis of pelletierine, two new 3-oxoglutaric acid synthesizers Type III PKSs, HsPKS4, and PcPKS1 of *Huperzia serrata*, and *Phlegmariurus cryptomerianus*, respectively, have been proposed to be involved in LA biosynthesis. HsPKS4 and PcPKS1 carry out the biosynthesis of pelletierine by providing 3-oxoglutaric acid which further undergoes a Mannich-like condensation with the Δ^1^-piperdeine starter substrate derived from lysine (Figure 7G). Interestingly, another latest finding has identified a metabolic regulon responsible for the synthesis of HupA in the club moss *Phlegmariusus tetrastichus*. The study demonstrated a developmentally controlled transcriptional coregulation of six enzymes including a Type III PKS and three Fe (II)/2-oxoglutarate-dependent dioxygenase (2-OGD) involved in the biogenesis of HupA (Nett et al., 2021). This study is the first to highlight the pathway leading to the synthesis of Lys-derived alkaloids through a tightly coordinated expression of secondary metabolic genes for the biosynthesis of medicinally important LAs.

## Concluding Remarks

Type III PKSs remains one of the most comprehensively studied enzyme systems and different in-depth reviews on the specificity, mechanical, potential, and structure-functional analysis of these simpler enzymes in synthesizing complex chemical scaffolds have been previously reported. Unarguably the said chemical diversity stems from a superfamily of enzymes that share a higher percentage (60%–75%) of their amino acid sequences. However, the enzyme family is equipped to create the chemodiversity through subtle changes in the active site cavity to accommodate a variety of starter substrates and to facilitate a variety of modes of cyclization and rounds of extension of the growing polyketide intermediate. This is the first review to discuss the different biological, medicinal and pharmaceutical properties of secondary plant metabolites derived from Type III PKS together with commenting upon their *in planta* functions. We also discussed briefly several mutagenesis studies that examine the starter specificities, the number of elongations, and mode of cyclization that affect the biochemistry and product profile of these enzyme systems. The review attempts to direct attention of readers to the remarkable promiscuity of the Type III PKS enzyme family in generating biologically active metabolites and lead drug molecules. Moreover, these enzyme systems hold enormous potential for bioengineering purposes to design unnatural natural polyketides with improved yield and activities.

## Author Contributions

PS and RB: conceptualization and visualization. RB, AB, and AS: literature search. RB, AB, and PS: writing and editing. All the authors proofread the manuscript.

## Funding

PS would like to acknowledge South Asian University Start-up grant, Innovative Young Biotechnologist Award (IYBA), Department of Biotechnology (DBT), Core Research Grant (CRG) (CRG/2018/002229), Science and Engineering Research Board (SERB), Department of Science and Technology (DST), and Government of India for financial support.

## Conflict of Interest

The authors declare that the research was conducted in the absence of any commercial or financial relationships that could be construed as a potential conflict of interest.

## Publisher's Note

All claims expressed in this article are solely those of the authors and do not necessarily represent those of their affiliated organizations, or those of the publisher, the editors and the reviewers. Any product that may be evaluated in this article, or claim that may be made by its manufacturer, is not guaranteed or endorsed by the publisher.
